# A Prospective Cohort Study on the Respiratory Effect on Modified Mallampati Scoring

**DOI:** 10.1155/2023/2193403

**Published:** 2023-08-23

**Authors:** Rotem Naftalovich, Marko Oydanich, Janet Adeola, Jean Daniel Eloy, Daniel Rodriguez-Correa, George L. Tewfik

**Affiliations:** ^1^Department of Anesthesiology, Rutgers New Jersey Medical School, Newark, NJ, USA; ^2^Medical Corps, U.S. Army, U.S. Army Medical Department, Fort Sam Houston, San Antonio, TX, USA

## Abstract

**Background:**

Mallampati scoring is a common exam method for evaluating the oropharynx as a part of the airway assessment and for anticipation of difficult intubation. It partitions the oropharynx into 4 categories with scores of 1, 2, 3, and 4. Even though its reliability is known to be limited by confounding factors such as patient positioning, patient phonation, tongue protrusion, and examiner variability, the effect of respiration, i.e., inspiration and expiration, has not yet been formally studied.

**Methods:**

Mallampati scores were collected from 100 surgical patients during both inspiration and expiration and later compared to the score obtained in the medical record, determined by a board certified anesthesiologist.

**Results:**

Score deviations from the medical record reference were compared for both inspiration and expiration showing that respiration affects Mallampati scores; for some patients, the scores improved (i.e., decreased), while in others they worsened (i.e., increased). The respiratory change effect was quantified and visualized by plotting the area under the curve of the histogram of the deviations. 42% of the patients had a worsening of scores by 1 or 2 points with inspiration while 36% of the patients had a worsening of scores by 1 or 2 points with expiration.

**Conclusions:**

Mallampati scoring is commonly used in evaluating the oropharynx as a part of the airway assessment and as a screening tool for difficult intubations. However, as this study points out, the respiratory cycle substantially affects the Mallampati scoring system, with significant deviations of 1 or 2 points. In a scoring system of 4 score categories, these deviations are remarkable.

## 1. Introduction

In 1983, Rao Mallampati first published a brief observational statement that large tongue volumes, which concealed the uvula, faucial pillars, and soft palate on examination of the oropharynx, were more likely to have a small angle between the tongue and the larynx rendering it difficult to access the larynx during intubation [1]. Mallampati stated in part “in the great majority of orotracheal intubation difficulties I have encountered … this clinical sign (concealment of faucial pillars and uvula by the tongue) was helpful in predicting intubation difficulty.” [1] Two years later, Mallampati published his findings concluding that the visibility of three pharyngeal structures, faucial pillars, soft palate, and uvula, could be used preoperatively as a proxy for laryngeal visualization during intubation [2]. It was then that Mallampati's three different classes were established. Class I represented visualization of all three pharyngeal structures; Class II represented visualization of only the faucial pillars and soft palate, and Class III only showing the soft palate. In 1987, Mallampati's class system was further updated by Samson and Young to include Class IV, a class which shows no visualization of any pharyngeal structures [3]. It is this modified classification that is commonly used for preoperative bedside airway evaluations in anesthesiology today.

The modified Mallampati class system has since become entrenched in the clinical airway assessment. The Mallampati score serves as a proxy in assessing how much physical space is available in the mouth for instrumentation during laryngoscopy and displacement of the tongue anteriorly. Given that an array of other clinical parameters affect the intubation process, it is not surprising that the reported sensitivity of the Mallampati score in identifying difficult intubations varies widely (from 11% to 84%) [4–7]. Furthermore, studies have even questioned its efficacy at predicting difficult intubation [8, 9]. Additionally, there are several variables that can affect Mallampati scoring that need to be taken into account (Table 1). Examiner variability is a consideration when using Mallampati [14]. Another is patient positioning, i.e., supine vs. sitting, which has been shown to influence Mallampati scores in predicting difficult tracheal intubations [10]. Tongue protrusion can also modify Mallampati scores [11]. Finally, patient phonation also influences Mallampati scoring by improving the predictability of difficult intubation [12, 15]. Because of its limitations, the Mallampati score has poor accuracy at identifying difficult intubations when used as the sole metric [9]. Nonetheless, Mallampati scores continue to be a very commonly used metric so there is value in identifying further limitations.

The pharyngeal airway is dynamic and changes shape and size during the respiratory cycle through inspiration and expiration [16, 17] such that the pharyngeal cross-sectional area increases during early expiration and decreases during late expiration with a net result of pharyngeal collapse throughout expiration [17, 18]. This is explained by increased activity of upper airway dilator muscles, particularly, the genioglossus, during inspiration and less activation of these muscles during expiration [17]. The activation of these muscles prevents airway collapse by countering the negative pressure generated in the pharynx during inspiration [17–20].

For that reason, in the current investigation, we investigated the effect of respirations on Mallampati scores. To test the hypothesis, we analyzed and compared Mallampati scores in the same patients during inspiration and expiration to determine the effect of the respiratory cycle on this scoring system.

## 2. Materials and Methods

### 2.1. Ethics Approval

A trained member of the anesthesia team conducted the airway assessments on surgical patients at University Hospital in Newark, New Jersey, USA. University Hospital is a 519-bed academic medical center with an active medical staff of more than 785. It is a Level I Trauma center in the state of NJ and a tertiary care center, often acting as a referral center from neighboring hospitals. It mainly cares for its local community of Newark, NJ, an underserved, racially diverse, impoverished community. The hospital performs about 12,000–13,000 surgeries per year. Since this study posed no invasive risk to any of the patients, the study was classified by the Newark Health Sciences Institutional Review Board as “exempt” from review. Verbal informed consent was obtained from each patient included in this study.

### 2.2. Conduct of the Study

A total of 100 surgical patients were included in this study. All patients undergoing surgery at University Hospital in Newark, New Jersey, were included in this study. The majority of the patients included in this study underwent orthopedic, neurosurgical, endoscopic, plastic, or cardiovascular surgeries/procedures. Children, pregnant women, prisoners, and non-English speakers were excluded from the study. Airways evaluations were based on the Mallampati methodology described in the literature and outlined briefly below [1, 3]. Patients were instructed to sit upright in the hospital bed or stretcher with their torso at a 90-degree angle. With the patient's head in the neutral position, patients were instructed to open their mouth as wide as possible and maximally protrude their tongue. While the patient's tongue remained protruded, patients were instructed to inhale and hold their breath while the inspiration Mallampati score was obtained. Then, in the same view, patients were instructed to exhale and the expiration Mallampati score was obtained. The examiner was positioned opposite the patient so that their eye level was at the level of the patient's oropharynx which was then visualized with a penlight. The airway was classified based on the modified four-class Mallampati scoring system, and the results were documented. The baseline Mallampati score, the score documented by the anesthesia team, was used as a reference to which both the inspiration and expiration scores of the same patient were compared to. Score deviations from the reference were considered positive when clinically more reassuring and closer to a score of 1, or negative when less reassuring and closer to a score of 4. For example, if a patient's reference Mallampati score was 2, but was 3 during inspiration, we would score that change as −1, as the score went from 2 to a worse score of 3. Scores were then partitioned into the following seven categories: worse: −3, −2, and −1, same: 0, or better: 1, 2, and 3. A histogram was then generated for both inspiratory and expiratory scores, and the area under the curve (AUC) was quantified. A similar analysis was conducted for the obese patients' subset (BMI > 30; *n* = 36).

### 2.3. Statistical Analysis

Demographic data were stratified for age, race, sex, BMI, and insurance status, and Chi-square analysis was used to determine differences in the population data.

## 3. Results

### 3.1. Sample Demographics

Sampled patients were mainly middle-aged with the majority falling within the age range of 40–59 years old (Table 2; *p* < 0.001). Biological sex was equally split, with 50% male and 50% female patients (*p* = 0.68). Race was predominantly Hispanic and Black (72%, *p* < 0.001). The majority of patients had Medicare insurance and had a BMI of 25–29.9. A significant portion of the population was obese (36%) with roughly 8% being morbidly obese (BMI > 40).

### 3.2. The Effect of Respiration on Mallampati Scoring

**Figure 1:**
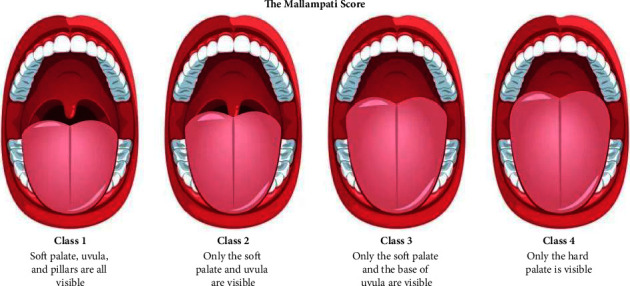
An illustrative summary of the different Mallampati scoring classes and how they are determined.

**Figure 2:**
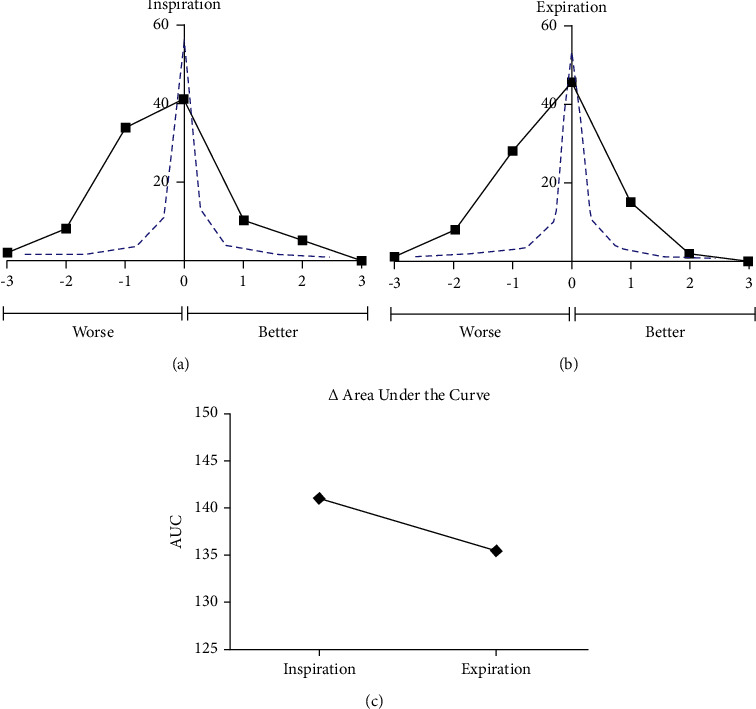
The change in score between the overall score and the inspiration (a) and subsequently the expiration (b) scores was plotted (black line). Both were compared to an ideal curve showing no difference between scores regardless of respiration statues (blue line). The area under curve (AUC) for both inspiration and expiration was calculated and compared showing an overall decrease in AUC when transitioning from inspiration to expiration (c).

A review of the Mallampati score classes is shown in Figure 1, giving a brief description of each class (I–IV). The change from each patient's reference medical record score to the score either under inspiration (Figure 2(a)) or expiration (Figure 2(b)) was plotted using a histogram and indicated by the black line. If Mallampati scores were independent of respiration, the expected change between the inspiratory, expiratory, or the medical record score would be minimal as approximated by the blue line. However, 42% of the patients had a worsening of scores by 1 or 2 points with inspiration while 36% of the patients had a worsening of scores by 1 or 2 points with expiration. The observed respiratory effect on Mallampati scores can also be appreciated when measuring the AUC of both respiration plots (Figure 2(c)). The AUC decreases from 141, during inhalation, to 135.5 during expiration.

### 3.3. Respiratory Effect on Mallampati Scores of Obese Patients

**Figure 3:**
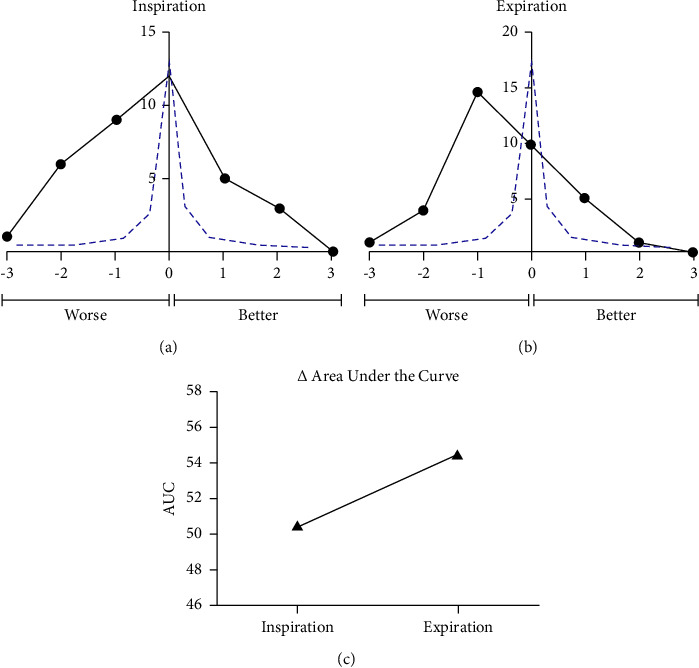
A subset of obese patients from the total study population was examined. The change in score between the overall score and the inspiration (a) and subsequently the expiration (b) scores was plotted (black line) and compared to the ideal curve in which Mallampati is unaffected by respiratory status (blue line). The AUC was calculated, showing an increase in the AUC when transitioning from inspiration to expiration (c).

From the sample, 36 patients were obese. Similar to the total study population, the change from the overall score for inspiration (Figure 3(a)) and expiration (Figure 3(b)) were plotted (*black line*) showing significant deviation from the ideal curve (*blue line*). Areas under the curve for both inspiration and expiration were measured. AUC increased from 50.5 during inhalation to 54.5 during expiration (Figure 3(c)).

## 4. Discussion

To our knowledge, this is the first study to demonstrate a change in Mallampati scores through the respiratory cycle. A scoring system of 4 scores with deviations of 1 or 2 score points can only be of limited value. The portion of deviations is remarkable; 42% of the patients had worsening of scores by 1 or 2 points with inspiration, while 36% of patients had a worsening of scores by 1 or 2 points with expiration.

When plotting the Mallampati scores during inspiration or expiration in comparison to those scores in the medical record, we noticed a significant change (Figures 2(a) and 2(b)). This is made especially apparent when the comparison is made to the null hypothesis (*blue line*) in which respiration does not affect Mallampati scoring. The following data helps appreciate this effect: 44% of the patient population had a worsening of the Mallampati score with inspiration and 37% of the patient population had a worsening of the Mallampati with expiration. 15% of the population had improvement of Mallampati score with inspiration, while 17% had improvement of the Mallampati score with expiration.

Worsening (increase) Mallampati score with expiration, as observed in this study, may be explained by pharyngeal changes that occur during expiration [17]. Under normal physiology, the pharyngeal airway is up to three times more collapsible during expiration as compared to inspiration [17]. Furthermore, there is a complete closure of the upper airway during mid-expiration [17, 21, 22]. This increased collapsibility of the upper airway is due to the physiological decrease in genioglossus muscle tone during expiration [17, 23] which, in turn, may lead to worsening of Mallampati scores. This muscle plays a key role in maintaining upper airway muscle tone and patency by countering two forces, the intraluminal negative pressure generated from inspiration and the extraluminal tissue pressure from surrounding structure [22, 24]. With regards to worsening of Mallampati scores with inspiration, it is possible that the weakening of this muscle could lead to airway collapse and manifest as a worse Mallampati score.

Obstructive sleep apnea (OSA) is a particular pathophysiology accompanied by dysfunction of upper airway muscles [25]. OSA patients have increased resistance to airflow in the oro-nasopharynx even when awake and exhibit disordered breathing patterns during sleep [25]. Interestingly, this increased resistance is even seen in OSA patients with normal, when compared to age controlled subjects, pharyngeal cross-sectional areas [25]. The etiology of this increased resistance is the dysfunction of the upper airway dilator muscles [25]. This dysfunction can lead to airway collapse during REM sleep which thereby further contributes to apneic episodes throughout the night. Not surprisingly, these patients who exhibit airway collapse with sleep-disordered breathing during REM also tend to have airway collapse during mask general anesthesia [26]. Patients who display worsening Mallampati score with inspiration may be more likely to have underlying OSA. In fact, it was even suggested that Mallampati scoring could be used to predict OSA presence and severity [13]. However, any such approach would have to take in to consideration the significant respiratory effect on Mallampati scoring.

In our studied obese patients, the respiratory change in Mallampati scores was more dramatic for both inspiration and expiration (Figures 3(a) and 3(b)) than that seen with the total sample of patients in the study. Specifically, 44% and 56% of obese individuals demonstrated worsening of Mallampati scoring during inspiration and expiration, respectively, and 22% and 16% showed improvement of Mallampati scores during inspiration and expiration, respectively. The difference between the entire patient sample and the obese subset is further exemplified when comparing the change in the AUC when transitioning from inspiration to expiration. While the AUC decreased from inspiration to expiration in the total patient population (Figure 2(c)), it actually increased in the obese group (Figure 3(c)), further suggesting the variability of Mallampati scoring with respiration and the added factor of obesity.

It is well established that obesity and the associated anatomical and physiological changes can alter proper air movement through the upper airway [27, 28]. The Mallampati score improves after bariatric surgery, suggesting that anatomical differences in the obese population affect respiration [28]. In this present study, the obese patient group had a higher percentage of worsening of Mallampati scores with expiration than the general study population. There are several anatomical factors in these patients that could account for this difference. Obese patients tend to have more soft tissue in the cervical neck and hypopharyngeal region, and this can contribute to the collapse of the pharyngeal walls [28]. It can lead to gradual increases in the concavity of the posterior epiglottis which can be used as a qualitative measure to determine the chronicity of airway collapse in obese patients [29]. Additionally, the hyoid bone in obese patients is positioned lower than that of healthy controls [28, 30]. A lower position of the hyoid bone pushes the tongue vertically which obscures the view of several oropharyngeal structures [28]. All of these anatomical differences can contribute to increased airway collapse during expiration resulting in score worsening. The obese patients in this study also had a higher percentage of worsening of Mallampati scoring with inspiration. Since obesity is a risk factor for OSA, it is likely that at least some obese patients exhibit similar dysfunction of upper airway dilator muscles that OSA patients experience. This in turn can lead to weakening of this musculature and to distortion of the view of oropharyngeal structures and the airway.

The current study is not without limitations, most notably, the small sample size. While a larger sample size would have reduced any significant deviation, we believe that our sample size was large enough to prove our hypothesis, i.e., that there is a difference in Mallampati scoring with respiration. Another limitation was the demographics of our study population, which consisted of mainly middle-aged Black and Hispanic patients. While it is known that differences in scores exist between certain races, e.g., Asians have a higher Mallampati score than caucasians [31], it does not affect the integrity of the study because we strictly focused on change in score from baseline during inspiration and expiration. Therefore, baseline differences between races are irrelevant as we focused on absolute change from each patient's individual baseline.

## 5. Conclusions

To our knowledge, this is the first study to demonstrate variations in the modified Mallampati scoring system with inspiration and expiration. We demonstrated that most patients had a change in Mallampati score during the respiratory cycle. Specifically, most patients exhibited a worsening (increase) of Mallampati score with inspiration and expiration. The staggering variations in Mallampati scores in the obese patient population when compared to the general study population could potentially be due to the anatomical changes that can alter air movement in obese individuals. Overall, our study demonstrated that the modified Mallampati scoring system is greatly affected by respiration, leading to a worsening of scores in a significant proportion of the population.

## Figures and Tables

**Table 1:** Summary of variables that affect the Mallampati score.

Variables affecting Mallampati scores	Change in Mallampati scoring
Patient positioning (supine vs. sitting)	Supine: increases and sitting: decreases [10]
Tongue protrusion	Increases [11]
Phonation	Decreases [12]
Obstructive sleep apnea	Increases [13]
Evaluator differences	Increases or decreases [14]

**Table 2:** Demographic information with chi-squared analysis.

	*N*	%	*p* value
Age categories			
Age group 1 (0–19)	5	5.0	*p* < 0.001
Age group 2 (20–39)	29	29.00	
Age group 3 (40–59)	34	34.00	
Age group 4 (60–79)	32	32.00	
Age group 5 (80+)	0	0.00	
Gender			
Male	50	50.00	*p* = 0.68
Female	50	50.00	
Race			
White	19	19.00	*p* < 0.001
Black	32	32.00	
Hispanic	40	40.00	
Other	9	9.00	
Insurance status			
Medicare	20	20.00	*p* < 0.001
Medicaid	43	43.00	
Private	20	20.00	
Uninsured	12	12.00	
BMI			
<20	1	1.01	*p* < 0.001
20–24.99	24	24.24	
25–29.99	39	39.39	
30–34.99	17	17.17	
35–39.99	11	11.11	
40+	8	8.08	

Note: Data are presented as a total number of patients (*N*) and percent of population (%).

## Data Availability

The patient data used to support the findings of this study are restricted by the Newark Health Sciences IRB in order to protect patient privacy. Data may be available for researchers who meet the criteria for access to this confidential data. To request the data, the principal investigator of this study, Rotem Naftalovich, MD, can be contacted at naftalro@njms.rutgers.edu.

## Additional Points

*What Is Known*? (i) The Mallampati score serves as a proxy in assessing how much physical space is available in the mouth for instrumentation during laryngoscopy and displacement of the tongue anteriorly. (ii) Examiner variability, patient positioning, tongue protrusion, and phonation have all been shown to affect the Mallampati score. *What Is New?* (i) The effect of respiration on Mallampati scoring was examined in this study. (ii) Both inspiration and expiration can increase Mallampati scoring. (iii) The obese population may have more variation in Mallampati scoring with changes in respiration.
